# Glucosylglycerol phosphorylase, a potential novel pathway of microbial glucosylglycerol catabolism

**DOI:** 10.1007/s00253-024-13035-3

**Published:** 2024-02-16

**Authors:** Lin Cheng, Zhichao Zhang, Daling Zhu, Quan Luo, Xuefeng Lu

**Affiliations:** 1 College of Chemical Engineering and Materials Sciences, Tianjin University of Science & Technology, Tianjin, 300457 China; 2 Key Laboratory of Biofuels, Qingdao Institute of Bioenergy and Bioprocess Technology, Chinese Academy of Sciences, Songling Rd 189, Qingdao, 266101 China; 3 Shandong Energy Institute, Songling Rd 189, Qingdao, 266101 China; 4 Qingdao New Energy Shandong Laboratory, Songling Rd 189, Qingdao, 266101 China; 5 Marine Biology and Biotechnology Laboratory, Qingdao National Laboratory for Marine Science and Technology, Wenhai Rd 168, Qingdao, 266237 China

**Keywords:** *Marinobacter*, Glucosylglycerol, Compatible solute, Salinity, GH13_18

## Abstract

**Abstract:**

Glucosylglycerol (GG) is a natural compatible solute that can be synthesized by many cyanobacteria and a few heterotrophic bacteria under high salinity conditions. In cyanobacteria, GG is synthesized by GG-phosphate synthase and GG-phosphate phosphatase, and a hydrolase GGHA catalyzes its degradation. In heterotrophic bacteria (such as some *Marinobacter* species), a fused form of GG-phosphate phosphatase and GG-phosphate synthase is present, but the cyanobacteria-like degradation pathway is not available. Instead, a phosphorylase GGP, of which the coding gene is located adjacent to the gene that encodes the GG-synthesizing enzyme, is supposed to perform the GG degradation function. In the present study, a GGP homolog from the salt-tolerant *M. salinexigens* ZYF650^T^ was characterized. The recombinant GGP catalyzed GG decomposition via a two-step process of phosphorolysis and hydrolysis in vitro and exhibited high substrate specificity toward GG. The activity of GGP was enhanced by inorganic salts at low concentrations but significantly inhibited by increasing salt concentrations. While the investigation on the physiological role of GGP in *M. salinexigens* ZYF650^T^ was limited due to the failed induction of GG production, the heterologous expression of *ggp* in the living cells of the GG-producing cyanobacterium *Synechocystis* sp. PCC 6803 significantly reduced the salt-induced GG accumulation. Together, these data suggested that GGP may represent a novel pathway of microbial GG catabolism.

**Key points:**

• *GGP catalyzes GG degradation by a process of phosphorolysis and hydrolysis*

• *GGP-catalyzed GG degradation is different from GGHA-based GG degradation*

• *GGP represents a potential novel pathway of microbial GG catabolism*

**Supplementary Information:**

The online version contains supplementary material available at 10.1007/s00253-024-13035-3.

## Introduction

Exposure of microorganisms to high salinity conditions usually triggers a fast loss of intracellular water to extracellular environments, which in turn results in a decrease of cell turgor and affects the viability of cells. To counteract this influence, many microorganisms accumulate large amounts of compatible solutes, a functional group of organic compounds generally with low molecular weights, neutral net charge, and good biocompatibility (Brown 1976), in the cell by de novo synthesis and/or uptake from environments (Kempf and Bremer 1998). This strategy ensures basic survival and further growth of microbial cells upon salt stress. When the environmental salinity shifts to standard conditions, the levels of intracellular compatible solutes decline via catabolism or exudation to achieve a new balance of osmotic potential.

**Fig. 1:**
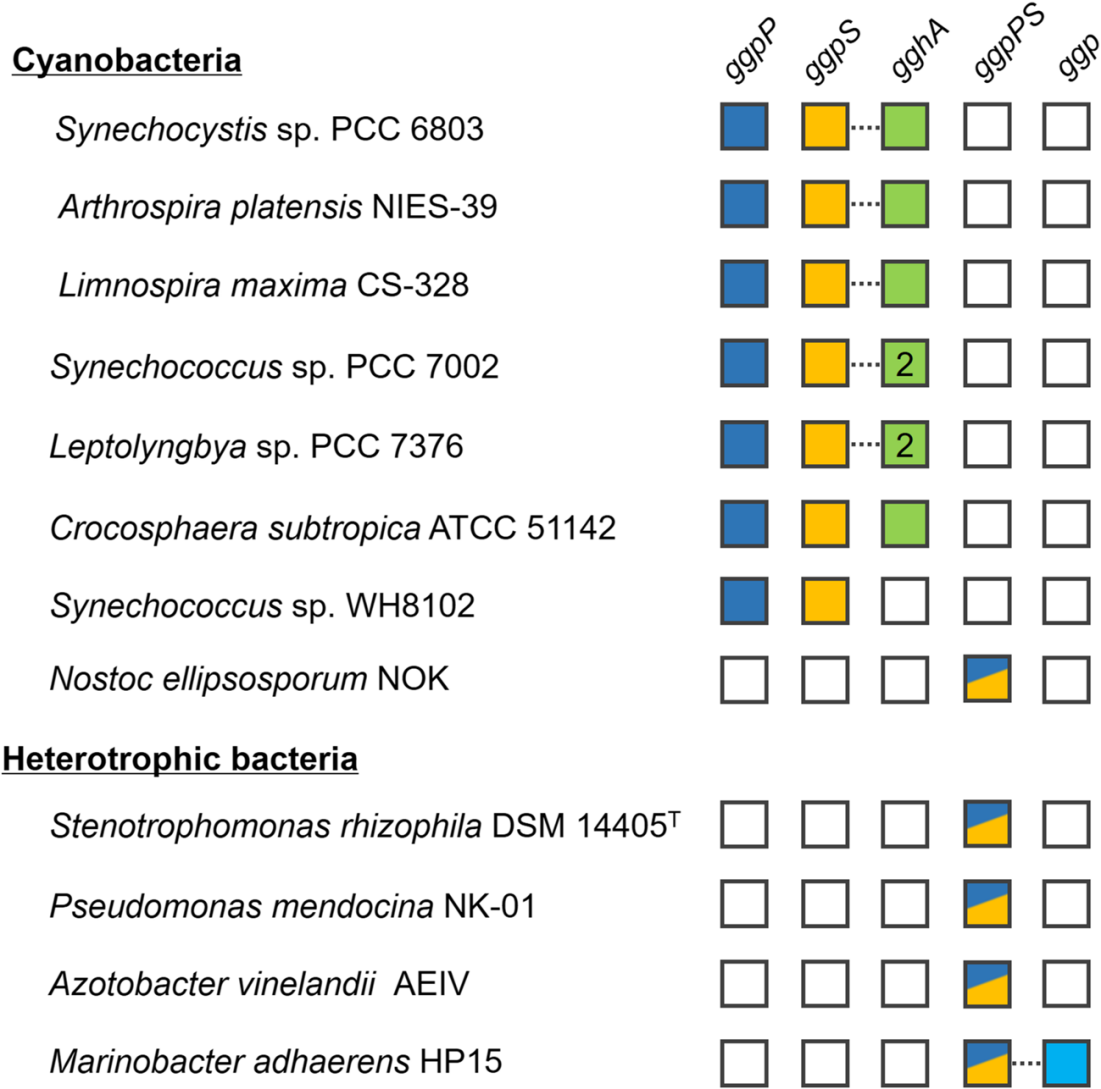
Illustration of the presence of the key genes participating in microbial GG metabolism in the genomes of the identified or potential GG-producing cyanobacteria and heterotrophic bacteria. *ggpS* and *ggpP* compose the GG-synthesizing pathway. *gghA* represents the GG-degrading pathway. *ggp* is the putative novel GG-degrading pathway investigated in the present study. The filled and open squares indicate the presence and absence of the target genes, respectively. The number “2” in the squares demonstrates that two target genes are present. The co-location of genes in the genomes is demonstrated by the dotted lines. The locus tags of *ggpP*/*ggpS*/*gghA* are *slr0746*/*sll1566*/*slr1670* for Syn6803, *NIES39_R01210*/*M02120*/*M02110* for *A. platensis* NIES-39, *AmaxDRAFT_4256*/*2350*/*2351* for *L. maxima* CS-328, *SYNPCC7002_A2841*/*A2851*/*A2843* + *A2849* for *Synechococcus* sp. PCC 7002, *Lepto7376_0796*/*0792*/*0793* + *0794* for *Leptolyngbya* sp. PCC 7376, and *Cce_3291*/*2391*/*3236* for *C. subtropica* ATCC 51142, respectively. The locus tags of *ggpP* and *ggpS* in *Synechococcus* sp. WH8102 are *SYNW0860* and *SYNW1281*, respectively. The locus tags of *ggpPS* are *JMG10_34610* for *N. ellipsosporum* NOK, *DX03_04600* for *S. rhizophila* DSM 14405^T^, *MDS_3416* for *P. mendocina* NK-01, *AVAEIV_003468* for *A. vinelandii* AEIV, and *HP15_2852* for *M. adhaerens* HP15, respectively. The locus tag of *ggp* in *M. adhaerens* HP15 is *HP15_2853*

Among the compatible solutes (e.g., trehalose, sucrose, proline, 2-*O*-α-glucosyglycerol [GG], ectoine, and glycine betaine [GB]) identified in microorganisms (Dandapath et al. 2017; Kempf and Bremer 1998; Klähn and Hagemann 2011), GG attracts increasing interest from industry and academia in recent years because of its desirable properties/activities (such as clear-cut sweetness, noncariogenicity, excellent water-holding capacity, protective effects on macromolecules, and antitumor activity) and potential applications in the agronomy, health care, cosmetics, and pharmacy fields (Luo et al. 2022). Nowadays, GG is commercially produced by at least two methods (i.e., enzymatic synthesis and native extraction from cyanobacteria) and has been widely used as an excellent moisturizer in many cosmetic and personal care products (Luo et al. 2022). Concerning the basic research, the genetic/biochemical background and regulatory mechanisms of microbial (especially cyanobacterial) GG anabolism have been elucidated (Hagemann and Erdmann 1994; Mikkat et al. 1996; Novak et al. 2011). GG is synthesized by a two-step process. GG-phosphate synthase (GGPS) first catalyzes the conversion of ADP-glucose and glycerol-3-phosphate to GG-3-phosphate and ADP. A phosphatase, i.e., GG-phosphate phosphatase (GGPP), then hydrolyzes GG-3-phosphate to generate GG. The two enzymes are encoded by two separate genes *ggpS* and *ggpP* in nearly all GG-producing cyanobacteria (except *Nostoc ellipsosporum* NOK) (Fig. 1) (Hagemann et al. 1996, 1997; Marin et al. 1998). In some heterotrophic bacteria such as *Stenotrophomonas rhizophila* DSM14405^T^, *Pseudomonas mendocina* NK-01, *Azotobacter vinelandii* AEIV, and *Marinobacter adhaerens* HP15, the GGPS and GGPP candidates are also present, but they are encoded as a fused protein by a single gene (termed *ggpPS*) with GGPP as its N-terminal domain and GGPS as its C-terminal domain (Hagemann et al. 2008; Klähn and Hagemann 2011). This suggests possible differences of these bacteria from cyanobacteria in GG metabolism.

To finely regulate intracellular contents and avoid a net loss of carbon and energy, microbial cells are generally thought to recycle compatible solutes when the stress environment shifts to normal. The molecular basis of microbial GG catabolism was recently unraveled in the model cyanobacterium *Synechocystis* sp. PCC 6803 (hereafter Syn6803) (Kirsch et al. 2017; Savakis et al. 2016). A glycoside hydrolase GGHA, encoded by *slr1670*, catalyzed the hydrolysis of GG, generating glycerol and glucose. As predicted, putative *gghA* genes are widely found in the GG-producing cyanobacteria (Fig. 1). In a few species such as *Synechococcus* sp. PCC 7002 (hereafter Syn7002) and *Leptolyngbya* sp. PCC 7376, even two *gghA* homologs are available. Intriguingly, in most cases, the *gghA* gene is co-located with the *ggpS* gene, which supports its functional correlation. This organization pattern may facilitate efficient synergistic regulation of GG anabolism and catabolism. However, the homologs of *gghA* are not found in the heterotrophic bacteria possessing the GGPPS pathway (Fig. 1), remaining the mechanism of GG catabolism mysterious in these microorganisms.

Recently, we noticed that the genome of the halotolerant bacterium *M. adhaerens* HP15 harbors a *ggpPS* gene (*HP15_2852*) that should enable GG synthesis via a fusion enzyme showing synthase and phosphatase activity as well as a putative hydrolase gene (*HP15_2853*) that is located downstream of *ggpPS* (Fig. 1) (Luo et al. 2022). Considering their predicted functions and genomic organization, we supposed that the hydrolase is involved in GG catabolism. Because of the low sequence identity (13%) to GGHA, the HP15_2853 protein may have a different behavior from GGHA. The results of Franceus and colleagues indicated that *HP15_2853* codes for a glycoside hydrolase (termed GGP) belonging to the subfamily 18 of glycoside hydrolase family 13 (GH13_18) (Franceus et al. 2018). In the present study, the function of GGP was further determined with a homolog from *M. salinexigens* ZYF650^T^ by both biochemical characterization and physiological analyses. We found that this GGP catalyzed GG decomposition via a two-step process of phosphorolysis and hydrolysis in vitro. The *ggp* genes were not only found in *M. salinexigens* and *M. adhaerens*, but also present in many members of the *Marinobacter* genus. It may represent a novel pathway of microbial GG catabolism.

## Materials and methods

### Strains and cultivation conditions

*M. salinexigens* strain ZYF650^T^ (MCCC 1K03552^T^) was provided by Marine Culture Collection of China. *Marinobacter* cells were grown in 2216E medium or modified marine broth (MB: 5 g/l peptone, 1 g/l yeast extract, 0.04 g/l ferric citrate) at 30 ℃, and different concentrations of NaCl were supplemented as indicated in the text. Syn6803 was from the strain collection of our lab. For standard cultivation, Syn6803 cells were grown in BG11 medium aerated with sterile air at 30 °C under constant white-light illumination of 120 μmol/m^2^/s. To induce GG production, cyanobacterial cells cultivated under the standard condition were transferred into BG11 medium containing 4% NaCl. *Escherichia coli* cells were grown in lysogeny broth (LB) at 37 °C. Cell growth was monitored by measuring the optical density at 600 nm (OD_600_, for *Marinobacter* and *E. coli*) or 730 nm (OD_730_, for Syn6803). Antibiotics such as chloramphenicol (Cm, 5 [in MB] or 20 μg/ml [in 2216E] for *Marinobacter*, 10 μg/ml for Syn6803) and kanamycin (50 μg/ml for *E. coli*) were applied when required. For solid medium, 1.6% (w/v) agar was supplemented. All quantitative data were presented as means from three independent replicates.

### Sequence analyses

The protein datasets of 250 *Marinobacter* isolates (supplementary information Table S1) were obtained from the “Genome” database of NCBI (https://www.ncbi.nlm.nih.gov/) using the integrated program Batch Entrez. Blastp analyses were conducted in a local manner using BLAST + (version ncbi-blast-2.13.0 +), and the threshold of outputs was set at the sequence identity of 30%. The software Origin was used to generate heatmaps. The phylogenetic relation of GGP proteins was analyzed with the software MEGA using the neighbor-joining method.

### Analyses of intracellular compatible solutes

The extraction and determination of intracellular compatible solutes (mainly GG) of Syn6803 were performed as previously described (Qiao et al. 2019). The same procedure was applied to *Marinobacter*.

### Protein expression and purification

The coding sequence (*FWJ25_14990*) of *M. salinexigens* ZYF650^T^ GGP was synthesized by BGI (Beijing, China) after codon optimization (supplementary information), and cloned into pET-28b vector with an N-terminal His-tag. To overexpress *ggp*, *E. coli* BL21(DE3) cells harboring the expression plasmid were induced by 0.2 mM isopropyl-β-D-thiogalactopyranoside for 20 h at 20 ℃. Centrifugation was conducted at 8228 × *g* for 20 min to harvest cells. Cell pallets were resuspended and disrupted by sonication in prechilled binding buffer (20 mM KH_2_PO_4_-K_2_HPO_4_, pH 7.0, 300 mM NaCl). The lysates were centrifuged at 8228 × *g* for 60 min. After filtration through 0.22-μm polyethersulfone membrane, the supernatants were applied to Ni–NTA affinity columns for protein purification. Non-specific proteins were removed with wash buffer (binding buffer containing 50–60 mM imidazole), and the target protein was obtained at an imidazole concentration of 200 mM. The removal of imidazole was conducted by ultrafiltration of 30-kDa, and the resulting GGP protein was stored in the binding buffer for enzyme assays. The purity of the target protein was examined by SDS-PAGE. The protein content was determined according to the Bradford method (Bradford 1976). For the phosphorolytic and hydrolytic assays, GGP was purified using 20 mM Tris–HCl (pH7.0, containing 300 mM NaCl) as the basic buffer, and the purification procedure was the same as above.

### Enzyme assays

All enzyme assays were conducted in 200-μl reaction mixtures. Initially the enzymatic degradation of GG by GGP was evaluated by incubating 20 mM GG and a proper amount of GGP in 20 mM PB buffer (KH_2_PO_4_-K_2_HPO_4_, pH7.0) at 30 ℃. Unless noted otherwise, further assays were conducted in the mixtures of 20 mM PB or MES buffer (as indicated in the text), 20 mM GG, and 0.05 mg/ml GGP at 45 ℃ for 0.5 h to determine enzyme activity. To determine the hydrolytic activity, 20 mM MES solution (pH7.0) was used as the reaction buffer, and 20 mM α-glucose-1-phosphate (αG1P) was used as the substrate. All reactions were terminated by heating at 90 ℃ for 10 min. The amounts of GG, glucose, and glycerol in reactions were determined using an ICS5000/5000^+^ ion-exchange chromatography system (ThermoFisher, Waltham, MA, USA) as previously described (Qiao et al. 2018). The content of αG1P was determined using a αG1P assay kit (Grace Biotechnology, Suzhou, China). All quantitative data were presented as means from three independent replicates.

### Construction of the *ggp*-deficient mutant of *Marinobacter*

All plasmids and primers used in the present study are listed in Table 1. To inactivate *ggp* in *M. salinexigen* ZYF650^T^, the Cm-resistant marker Cm^r^ was amplified from plasmid pQL199 employing primers Msa-Cm-F/Msa-Cm-R, and the flanking regions of *ggp* were amplified using *M. salinexigen* ZYF650^T^ genomic DNA and primers MsaGGPko-up-F/MsaGGPko-up-R and MsaGGPko-dn-F/MsaGGPko-dn-R. The three fragments were linked and cloned into the pCE-Zero vector using the ClonExpress® Ultra One-Step-Cloning Kit (Vazyme Biotech, Nanjing, China), yielding plasmid pCL8. *M. salinexigens* cells, cultivated in 2216E medium at 30 °C for 24 h, were harvested at 4 °C. The cells were washed twice with prechilled sucrose solution (300 mM) and resuspended in the same solution to a final cell density of OD_600_≈2.0. pCL8 was introduced into *M. salinexigens* cells by electroporation. Briefly, 100 μl of cells was mixed with 100 ng plasmid DNA and electro-pulse treated for 5 ms using a micropulser (Bio-Rad, CA, USA) with the following settings: 12.5 kV/cm, 25 μF, and 200 Ω. Immediately after pulse, the cells were supplemented with 900 μl of 2216E medium and regenerated at 30 °C for 20 h by shaking. The cells were plated on 2216E agar plates containing 20 μg/ml Cm. Resistant transformants were obtained after 24 h of selection. The genotypes of the transformants were verified by PCR.

**Table 1:** Strains, plasmids, and oligonucleotides used in the present study

Strain, plasmid, or oligonucleotide	Characteristic, description, or sequence (5′–3′)	Reference or source
Plasmid		
pQL199	Harbors a Cm^r^ cassette, Cm^r^	Our lab
pJS40	Harbors a P_cpc560_-drived expression cassette in *slr0168* platform, Cm^r^	Our lab
pCL8	For the construction of the *M*. *salinexigens* Δ*ggp* mutant, Cm^r^	This study
pCL10	Harbors the Cm^r^-P_cpc560_-*ggp* fragment with the *gghA* flanking regions, Cm^r^	This study
pCL11	For the construction of the Syn6803 Δ*gghA* mutant, Cm^r^	This study
pCL12	Harbors the Cm^r^-P_cpc560_-*ggp* fragment with the *slr0168* flanking regions, Cm^r^	This study
pCL14	Harbors the Cm^r^-ENYC4-*ggp* fragment with the *gghA* flanking regions, Cm^r^	This study
Oligonucleotide		
Msa-Cm-F	CGAATAAATACCTGTGACGG	
Msa-Cm-R	TCTGCCATTCATCCGCTT	
MsaGGPko-up-F	GGATCTTCCAGAGATCCTACGAGCGAATGCTGAACG	
MsaGGPko-up-R	CCGTCACAGGTATTTATTCGGTCATCCCAGGTGCCAAAG	
MsaGGPko-dn-F	AAGCGGATGAATGGCAGATTCACCGGCAAGTACGACC	
MsaGGPko-dn-R	CTGCCGTTCGACGATGAGCGTCATAGAAGGTGCAG	
Trbcl-ggp-F	CATATGAGGCCTAATCTAGAT	
Pcpc560-ggp-R	TGAATTAATCTCCTACTTGA	
ggp-Pcpc560-F	TCAAGTAGGAGATTAATTCAATGCTGCTCAAAAATGCAGT	
ggp-Trbcl-R	ATCTAGATTAGGCCTCATATGTCAGAACTCGAGGTCCCGA	
Cm-gghA-F	ATGCCTGGTTACGCCCCGCC	
ggp-gghA-R	TCAGAACTCGAGGTCCCGAG	
gghA-up-F	AATTCGGATCTTCCAGAGATACCCAAGTTAATTCCCGCCG	
gghA-Cm-up-R	GGCGGGGCGTAACCAGGCATTCCCAACGAAACAAGCCAGT	
gghA-ggp-F	CTCGGGACCTCGAGTTCTGAAAGGATTGAATTGCCGCAAC	
gghA-dn-R	TTCAACTGCCGTTCGACGATCTGATGGTTCAAATCCTGGC	
Cm-gghA-R	GATCCTACCTGTGACGGAAG	
gghA-Cm-dn-F	CTTCCGTCACAGGTAGGATCAAGGATTGAATTGCCGCAAC	
gghA-ENYC4-F	ATGCTGCTCAAAAATGCAGT	
gghA-ENYC4-R	ATCGGATCCTACCTGTGACG	
Cm-ENYC4-F	CGTCACAGGTAGGATCCGATAAATATTCTGAAATGAGCTG	
ENYC4-ggp-R	ACTGCATTTTTGAGCAGCATCTTGTTGCCTCCTTAGCAGG	

### Construction of recombinant Syn6803 strains

To overexpress *ggp* in the neutral site *slr0168* of Syn6803, an expression platform, which harbors Cm^r^, the strong cyanobacterial promoter P_cpc560_ (Zhou et al. 2014) and the flanking regions of *slr0168*, was amplified from plasmid pJS40 employing primers Trbcl-ggp-F/Pcpc560-ggp-R. The open reading frame (ORF) of *M. salinexigens* ZYF650^T^ *ggp* was amplified with primers ggp-Pcpc560-F/ggp-Trbcl-R. The two fragments were linked by One-Step-Cloning (see above) to generate plasmid pCL12_._ To overexpress *ggp* at a *gghA*-deficient background of Syn6803, a Cm^r^-P_cpc560_ fragment was amplified from pJS40 with primers Cm-gghA-F/Pcpc560-ggp-R. The *ggp* gene was amplified as above using primers ggp-Pcpc560-F/ggp-gghA-R, and the flanking regions of *gghA* were amplified from Syn6803 using gghA-up-F/gghA-Cm-up-R and gghA-ggp-F/gghA-dn-R. The four fragments were linked and cloned into pCE-Zero, yielding plasmid pCL10. In both pCL10 and pCL12, the *ggp* gene was under the control of P_cpc560_. To construct a *gghA-*deficient control, the Cm^r^ fragment (amplified by Cm-gghA-F/Cm-gghA-R) and the *gghA* flanking regions (amplified by gghA-up-F/gghA-Cm-up-R and gghA-Cm-dn-F/gghA-dn-R) were combined and cloned into pCE-Zero, yielding plasmid pCL11. For the inducible expression of *ggp*, the promoter P_cpc560_ of pCL10 was replaced by a theophyllin-regulated riboswitch ENYC4 (Qiao et al. 2018). The ENYC4 and vector fragments were amplified by Cm-ENYC4-F/ENYC4-ggp-R and gghA-ENYC4-F/gghA-ENYC4-R, respectively. After one-step-cloning, pCL14 was generated.

The final constructs, pCL10, pCL11, pCL12, and pCL14 were transferred to the wild-type strain of Syn6803 by natural transformation. The Cm^r^ transformants were obtained after at least 7 days of selection on BG11 agar plates. The genotypes of the transformants were verified by PCR. Recombinant strains QL513, QL514, QL515, and QL518 were obtained.

## Results

### Potential GG metabolism in *Marinobacter* by sequence analyses

**Fig. 2:**
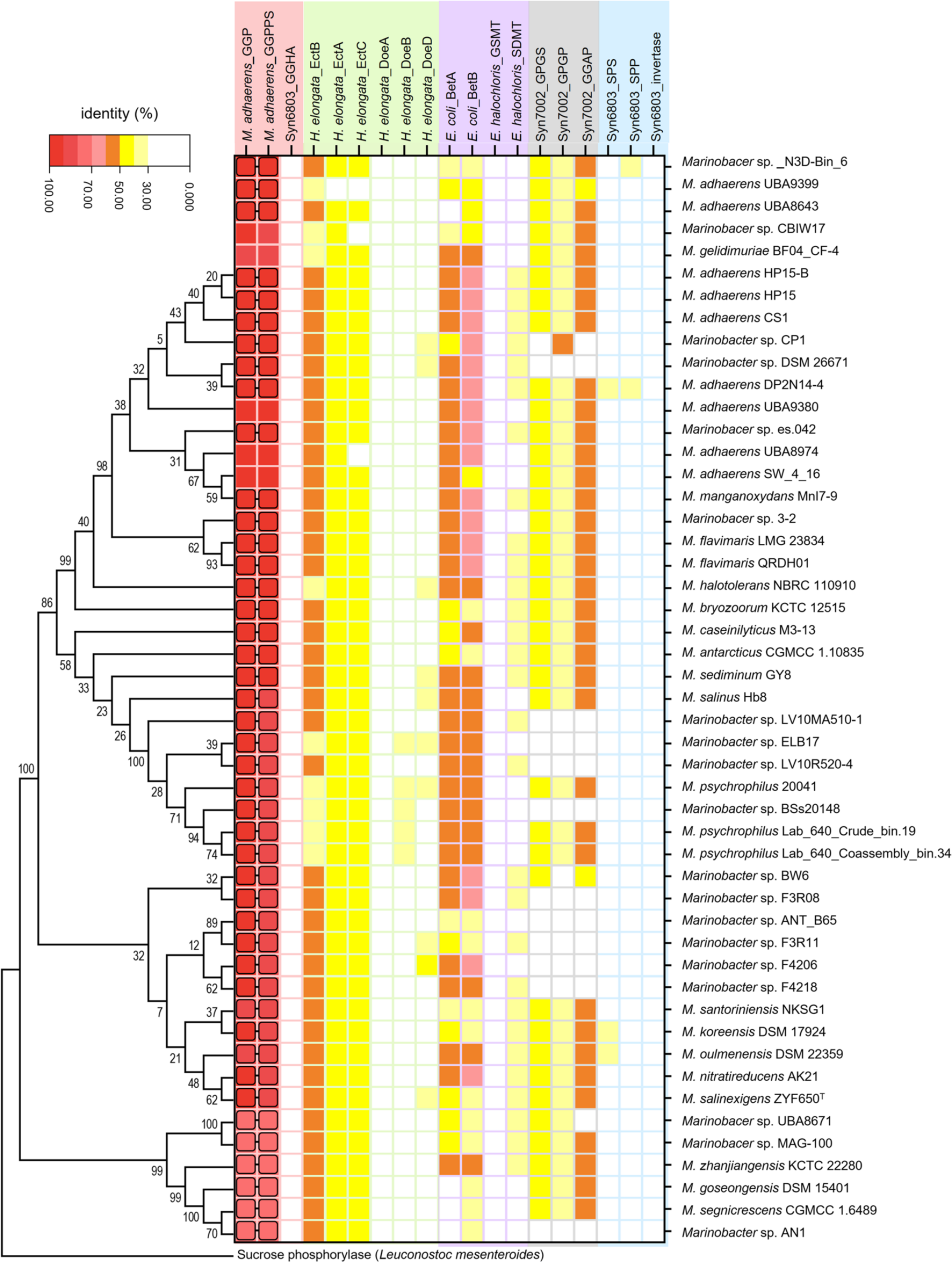
A heatmap of the putative proteins involved in microbial GG (pink area), ectoine (pale green area), GB (pale purple area), GGA (gray area), and sucrose (pale blue area) metabolisms in 49 *Marinobacter* isolates. The query sequences used for the local blastp analyses are given on the top. GGP (HP15_2853) and GGPPS (HP15_2852) are from *M. adhaerens* HP15. GGHA (Slr1670), SPS (Sll0045), SPP (Slr0953), and invertase (Sll0626) are from *Synechocystis* sp. PCC 6803. EctB (HALO_2589), EctA (HALO_2588), EctC (HALO_2590), DoeA (HALO_3665), DoeB (HALO_3664), and DoeD (HALO_3661) are from *H. elongate* DSM 2581^T^. BetA (CAA37093.1) and BetB (CAA37092.1) are from *E. coli*. GSMT (AAF87202.1) and SDMT (AAF87203.1) are from *Ectothiorhodospira halochloris*. GPGS (SYNPCC7002_A2021), GPGP (SYNPCC7002_A2023), and GGAP (SYNPCC7002_A2022) are from *Synechococcus* sp. PCC 7002. The frames and connecting lines on GGPs and GGPPSs indicate their adjacent gene organization. The phylogenic relation of 44 GGPs, which have complete ORFs, is presented on the left and *L. mesenteroides* sucrose phosphorylase (BAA14344.1) was used as a reference

To determine the occurrence of potential GG metabolism in the genus *Marinobacter*, local blastp analyses of GGP (HP15_2853) and GGPPS (HP15_2852) of *M. adhaerens* HP15 were performed against 250 annotated *Marinobacter* genomes (Table S1). The GGPPS homologs exhibiting ≥ 30% sequence identity were identified in 53 isolates, among which the GGP homologs were detected in 49 cases (Fig. 2). Despite annotated as sucrose phosphorylases that belong to the GH13_18 family, these GGP homologs were classified into a different clade from the well-characterized sucrose phosphorylase of *Leuconostoc mesenteroides* (Goedl et al. 2007), in the phylogenic analysis (Fig. 2). Excluding five isolates (*Marinobacter* sp. CBIW17, *M. gelidimuriae* BF04_CF-4, *M. adhaerens* UBA9380, *M. adhaerens* UBA8974, and *M. adhaerens* SW_4_16), in which the putative *ggpPS* or *ggp* genes occur at the ends of genomic contigs, the organization of the two genes in the genome is similar to that in *M. adhaerens* HP15, i.e., the *ggpPS* gene is located immediately upstream (17–76 bp) of *ggp* in the same direction. The search for the reported GG-degrading pathway, GGHA, was also performed. No homologs showing ≥ 30% sequence identity were identified, indicating that *Marinobacter* may have a different GG catabolic pathway from that of cyanobacteria.

The metabolic pathways of ectoine, GB, glucosylglycerate (GGA), and sucrose, the other four well-identified compatible solutes in bacteria (Costa et al. 2007; da Costa et al. 1998; Kirsch et al. 2019; Klähn et al. 2010), were additionally surveyed in the above 250 genomes (Table S1 and Fig. 2). The homologs (≥ 30% identity) of diaminobutyrate-2-oxoglutarate transaminase (EctB), diaminobutyrate acetyltransferase (EctA), and ectoine synthase (EctC), which compose the ectoine-synthesizing pathway of *Halomonas elongata* (Cánovas et al. 1998), were simultaneously identified in 228 *Marinobacter* isolates. No homologs of ectoine hydrolase (DoeA), the key enzyme involved in the well-identified ectoine-degrading pathway DoeABD (Schwibbert et al. 2011), were found in these isolates. In the case of GB synthesis, the *E. coli*-like BetAB pathway, catalyzing GB synthesis via choline oxidation by choline dehydrogenase (BetA) and betaine dehydrogenase (BetB) (Lamark et al. 1991), were detected in most of the *Marinobacter* isolates (i.e., 229 out of 250), whereas the de novo pathway consisting of glycine-sarcosine methyltransferase (GSMT) and sarcosine-dimethylglycine methyltransferase (SDMT) seemed not present in *Marinobacter* (Nyyssola et al. 2000). Regarding GGA, the homologs (≥ 30% sequence identity) of glucosyl-3-phosphoglycerate synthase (GPGS) and glucosyl-3-phosphoglycerate phosphatase (GPGP), which are responsible for GGA synthesis (Klähn et al. 2010), and the possible GGA-degrading enzyme GGAP (GGA phosphorylase) (Franceus et al. 2017), were simultaneously identified in 198 isolates. These results suggested that ectoine, GB, and GGA may be also employed as compatible solutes by *Marinobacter* for salt acclimation. On the other hand, only a few homologs of the sucrose-synthesizing enzymes, SPS and/or SPP, were found, and no homologs of the sucrose-degrading enzyme invertase were identified. Thus, the genus *Marinobacter* seems not to have the complete pathway of sucrose metabolism.

### Preliminary evidence of GG degradation by GGP

**Fig. 3:**
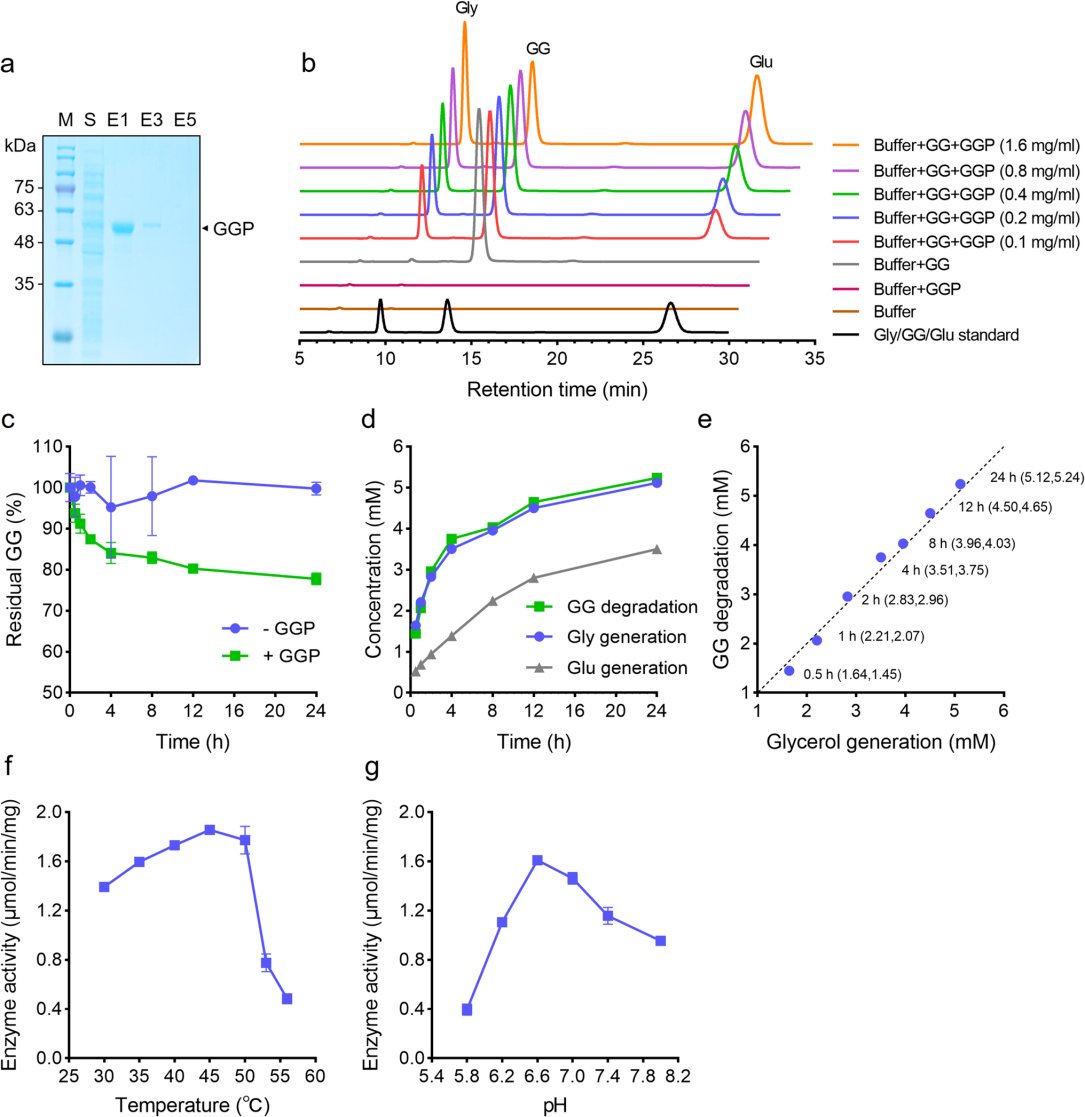
Analyses of the GG-degrading activity and other basic properties of GGP. **a** Examination of GGP purification by SDS-PAGE. M, protein marker; S, the supernatant fraction of cell extracts; E1–E5, different elution fractions at 200 mM imidazole. The solid triangle indicates the position of GGP in the gel. **b** The qualitative analysis of GG decomposition catalyzed by purified GGP. The assays were performed in PB buffer at 30 °C for 12 h with GG as the substrate. Gly, glycerol; glu, glucose. **c** Evaluation of the residual GG content in the presence ( +) or absence ( −) of GGP after different reaction times. The measured amounts were normalized by comparing them with the starting values (at 0 h). **d**, **e** The quantitative analysis of GG decomposition catalyzed by GGP (0.05 mg/ml). **f**, **g** The effects of reaction temperature and pH on the activity of GGP

To explore the GG-degrading activity, the putative *ggp* gene (*FWJ25_14990*) of *M. salinexigens* ZYF650^T^, a deep sea isolate from the Mariana Trench (Ahmad et al. 2020), was heterologously expressed in *E. coli* with an N-terminal His-tag. After Ni affinity chromatography, GGP was purified to apparent homogeneity. The SDS-PAGE as well as native PAGE analyses showed a single protein band with an estimated molecular mass of 57 kDa (Fig. 3a and Fig. S1), in agreement with its theoretical value (57.0 kD) deduced from the amino acid sequence.

**Fig. 4:**
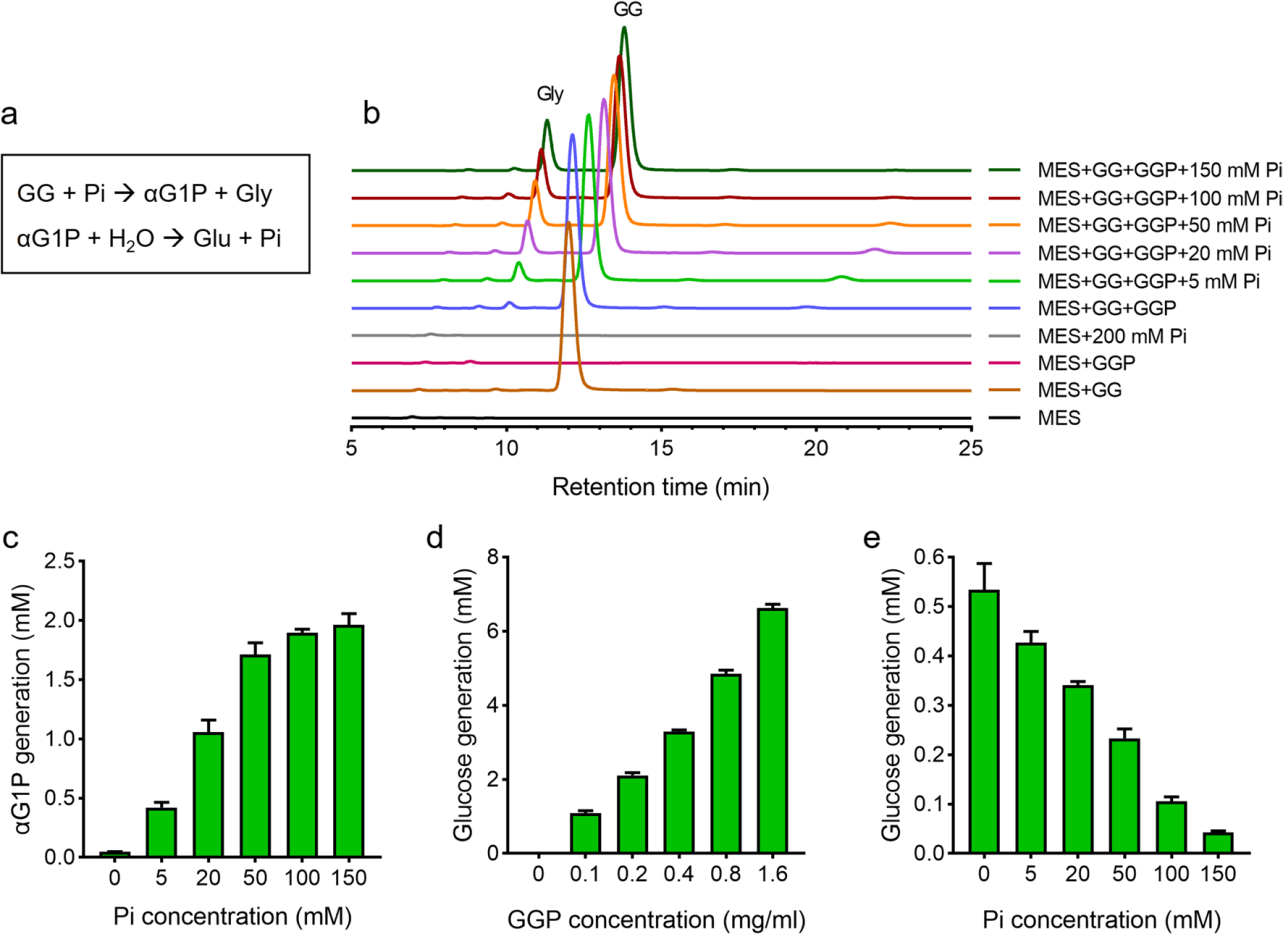
The proposed process of GG degradation by GGP (**a**) and biochemical verification of the phosphorolytic and hydrolytic steps during GG degradation (**b**–**e**). In **b** and **c**, the phosphorolytic assays were performed in 20 mM MES buffer (pH 6.6) with GG as the substrate. In **d** and **e**, the hydrolytic assays were performed in 20 mM MES buffer (pH 7.0) with αG1P as the substrate. A proper volume of PB buffer (pH6.6 or pH7.0) was supplemented to achieve the indicated Pi concentrations. Gly, glycerol; αG1P, α-glucose-1-phosphate; Glu, glucose

The degrading activity of GGP was examined using GG as the substrate (Fig. 3b). After 12-h incubation, two products, glycerol and glucose, were detected. The amounts of the products exhibited an elevated pattern with the increase of GGP amount, whereas the GG level gradually decreased. To exclude possible spontaneous decomposition, the stability of GG was analyzed in a time-course manner (Fig. 3c). In the absence of GGP, GG stayed on a relatively stable level within 24 h. However, the GG level declined rapidly within the first 4 h in the presence of GGP and was 77.8% of the initial level at 24 h. Further quantitative analyses showed that the amount of GG degradation matched well to that of glycerol generation but was clearly more than that of glucose generation (Fig. 3d and e), suggesting a two-step process of GG decomposition by GGP. Considering that GGP is predicted to be a phosphorylase of GH13_18 family, we supposed that it first phosphorolyzes GG to glycerol and αG1P and the latter compound is further hydrolyzed to produce glucose (Fig. 4a). Before we further verified this hypothesis, basic features of the enzyme were determined. GGP exhibited relatively high activity at temperatures of 30–50 °C and the highest activity was detected at 45 °C. At temperatures of above 50 °C, the activity rapidly declined (Fig. 3f). The optimal pH was pH6.6 (Fig. 3g). The thermostability of the enzyme was also determined by measuring the residual activity after heat treatment. The semi-inactivation temperature (*T*_50_^10^) was 53 °C and the half-life time at this temperature (*t*_1/2_) was around 8 h (Fig. S2).

### A two-step degradation of GG by GGP in vitro

To identify GG phosphorolysis, the PB buffer used in the above experiments was changed to MES buffer and a Pi gradient was supplemented (Fig. 4b). Elevated Pi concentrations caused improved generation of glycerol and αG1P (Fig. 4b and c), indicating a proportional increase of GG degradation with the elevated Pi level. To verify the subsequent hydrolytic reaction, αG1P was used as the substrate and the glucose product was determined. As predicted, the amount of glucose was elevated with the increase of GGP content (Fig. 4d) and reduced with the increase of Pi concentration (Fig. 4e). Taken together, these results confirmed that GG degradation catalyzed by GGP in vitro is a successive process of phosphorolysis and hydrolysis.

### Salt-mediated regulation of GGP activity and its substrate specificity

**Fig. 5:**
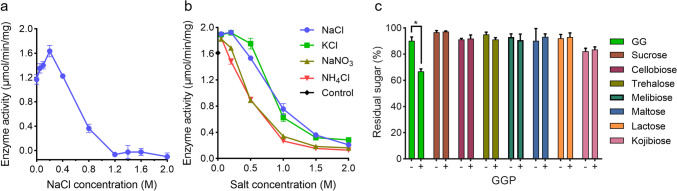
Effects of inorganic salts on the GG-degrading activity of GGP (**a**, **b**) and examination of the substrate specificity of GGP (**c**). In **a** and **b**, the assays were performed in 20 mM PB buffer (pH6.6) for 0.5 h with GG as the substrate. Different concentrations of NaCl, KCl, NaNO_3_, and NH_4_Cl were included in the reaction mixtures as indicated. The reaction without salt addition was used as the control. In **c**, the assays were performed for 12 h in the presence ( +) or absence ( −) of GGP with GG, sucrose, cellobiose, trehalose, melibiose, maltose, lactose, and kojibiose as the substrates, respectively. The obtained values were normalized by comparing them with the starting values (at 0 h). The asterisk indicates significant difference between the tested samples (Student’s *t* test, *P* < 0.01)

GG is known to function as a compatible solute for microbial salt acclimation. Its metabolism can be regulated on transcriptional, translational, and post-translational levels in cyanobacteria, and the salt-mediated modulation of the activities of the key enzymes (e.g., GGPS and GGHA) plays an important role for the fast response during acclimation (Kirsch et al. 2017; Marin et al. 2002; Novak et al. 2011). We supposed that GGP activity might be affected by salt concentrations. To this end, the impact of increasing inorganic salt concentrations on GGP activity was determined. Under the salt-free condition, GGP exhibited a noticeable activity of 1.17 μmol/min/mg (Fig. 5a). Although slightly activated at low concentrations (0–0.2 M), the enzyme activity was inhibited by the addition of NaCl (0.2–2.0 M). The activity declined to 22% (0.36 μmol/min/mg) of the highest level (1.63 μmol/min/mg, at 0.2 M) at 0.8 M NaCl and to almost non-detectable level at NaCl concentrations of 1.2 M or higher. The decreased activity seemed not due to the disruption of the protein structure. An impact of high salinity (up to 2 M NaCl) on protein status was not observed in the native PAGE analysis (Fig. S1). To explore whether such regulation exhibits salt preference, the impacts of additional three inorganic salts (i.e., KCl, NaNO_3_, and NH_4_Cl) were investigated in parallel with NaCl (Fig. 5b). Similarly, a strong inhibition, starting with a gentle activation at low salt concentrations (0–0.2 M for KCl, 0–0.05 M for NaNO_3_ and NH_4_Cl), by increasing salt concentrations was observed for all these salts. Thus, GGP exhibits a salt-dependent regulation of enzyme activity, similar to that of the previously identified GG- and sucrose-catabolizing enzymes, GGHA and invertase (Kirsch et al. 2018, 2017).

The substrate specificity of GGP was analyzed with seven disaccharides (sucrose, cellobiose, trehalose, melibiose, maltose, lactose, and kojibiose) in parallel with GG (Fig. 5c). After 12-h reaction, the residual amount of GG in the presence of GGP was apparently lower than that in the absence of the enzyme. Under the same condition, the residual amounts of other disaccharides were similar to the controls. These observations indicated a strict specificity of GGP toward GG.

### Effects of *ggp* inactivation in *M. salinexigens*

**Fig. 6:**
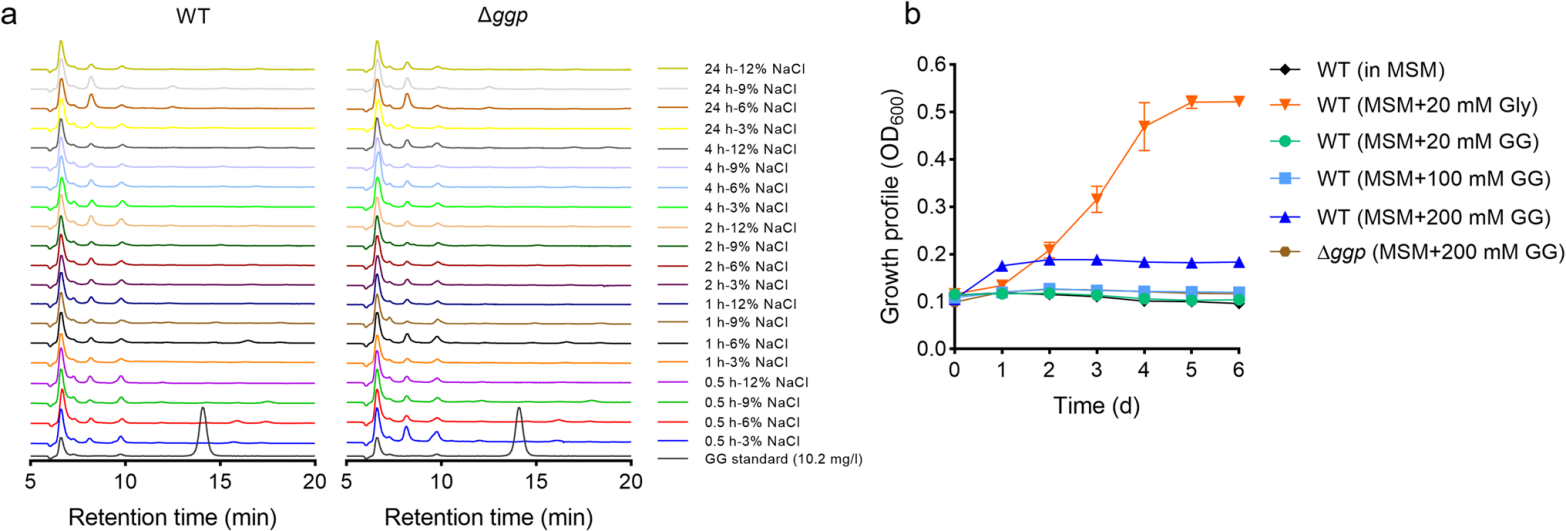
Analyses of the possible GG accumulation of the *M. salinexigens* ZYF650^T^ wild-type (WT) and Δ*ggp* strains under different NaCl concentrations (**a**) and the growth of the *M. salinexigens* ZYF650^T^ wild-type and Δ*ggp* strains with GG as the sole carbon and energy source (**b**). In **a**, the cells grown in MB medium containing 3% NaCl were transferred into the same medium with 3–12% NaCl. In **b**, cells were grown in minimal salt medium (MSM: 5.3 g/l MgCl_2_-6H_2_O, 0.75 g/l KCl, 0.1 g/l MgSO_4_-7H_2_O, 50 mg/l K_2_HPO_4_, 1 g/l NH_4_Cl, 0.74 g/l CaCl_2_-2H_2_O, 0.42 g/l NaHCO_3_, and 20 g/l NaCl) supplemented with 20 mM glycerol or 20–200 mM GG

Before the genetic manipulations in *M. salinexigens* ZYF650^T^, the salt tolerance and antibiotic sensitivity of the strain were determined. *M. salinexigens* ZYF650^T^ showed growth in the medium containing 3–12% NaCl (Fig. S3a), similar to that reported by Ahamad et al. (Ahmad et al. 2020). Under the NaCl-free and 15% NaCl conditions, no growth was observed. Whereas *M. salinexigens* ZYF650^T^ showed apparent resistances to kanamycin, apramycin, and neomycin (minimum inhibitory concentration > 100 μg/ml), high sensitivities to ampicillin and chloramphenicol were seen (Fig. S3b). To explore the GG-catabolizing activity of GGP in vivo, the *ggp* gene was inactivated (Δ*ggp*) by inserting a Cm^r^ cassette and the possible GG production was tracked (Fig. 6a). *Marinobacter* cells were cultivated in MB medium containing 3% NaCl and then treated by elevated NaCl concentrations (3–12%). Contrary to our expectation, GG was not detected in the wild-type cells at all NaCl concentrations during the whole testing period (0–24 h). No detectable GG production was observed in the Δ*ggp* cells as well under the same condition. Similar experiments were also performed in minimal salt medium with glycerol as the sole carbon and energy source. As seen above, no GG was accumulated by the wild-type and Δ*ggp* cells under elevated NaCl concentrations (Fig. S4a). The capability of *M. salinexigens* ZYF650^T^ to assimilate exogenous GG was also analyzed (Fig. 6b). Cells were grown in minimal salt medium with GG as the sole carbon and energy source. Glycerol was used as the positive control. At GG concentrations of 20 and 100 mM, the wild-type strain did not grow as the negative control (without carbon and energy source). A weak growth was observed at a relatively high GG concentration of 200 mM within the first 2 days. Under the same condition, the Δ*ggp* mutant did not grow at all.

### Effects of *ggp* expression in Syn6803

**Fig. 7:**
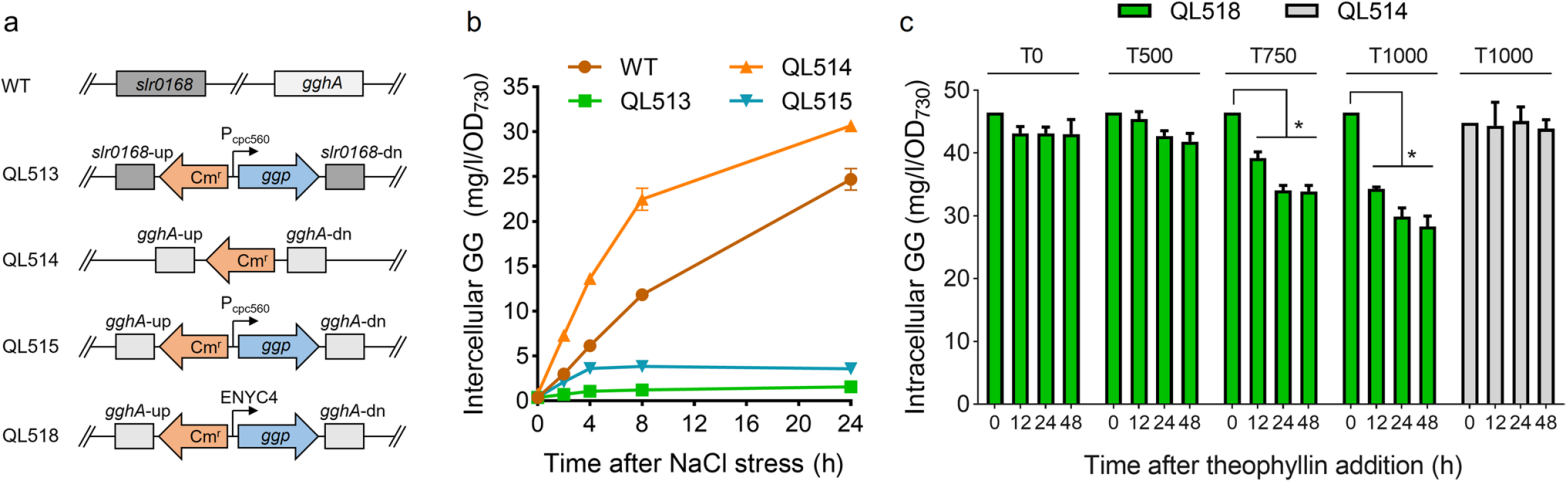
Analyses of the salt-induced GG accumulation of the wild-type (WT) and mutant strains of Syn6803. In **a**, the genotypes of the WT and mutant strains are illustrated. “up” and “dn” represent the upstream and downstream flanking regions of target genes, respectively. In **b**, the WT and mutant (QL513, QL514, QL515, and QL518) cells grown in the standard BG11 medium were stressed by 4% NaCl to induce GG production. In **c**, the cells of QL514 and QL518 were cultivated in BG11 medium containing 4% NaCl for 48 h and then supplemented with different concentrations of theophylline to induce *ggp* expression. T0, T500, T750, and T1000 represent 0, 500, 750, and 1000 μM theophylline, respectively. The asterisks indicate significant differences from the starting levels (0 h) (Student’s *t* test, *P* < 0.01)

The physiological function of *ggp* was further investigated in the GG-producing cyanobacterium Syn6803. The mutant strain QL513, expressing the *ggp* gene of *M. salinexigens* ZYF650^T^ under the control of a strong cyanobacterial promoter P_cpc560_ at the neutral site *slr0168*, was constructed (Fig. 7a). Syn6803 cells were exposed to 4% NaCl for GG production (Fig. 7b). As predicted, the wild-type Syn6803 exhibited a fast accumulation of GG within 24 h upon salt stress. Under the same condition, GG production of QL513 was greatly depressed. The GG content of the mutant was only 1.6 mg/l/OD_730_ in 24 h, which was much lower than the wild-type level (24.7 mg/l/OD_730_). Similar genetic construction was performed in the *gghA*-deficient background (QL514), generating QL515. Due to the inactivation of the native GG-catabolizing pathway, QL514 showed an enhanced GG accumulation compared with the wild-type. Like that in QL513, the production of GG was dramatically depressed by the expression of *ggp*.

In additional to the perspective of GG accumulation, GGP function was also analyzed from the perspective of GG degradation. Mutant QL518, in which the *ggp* gene was placed under the control of the theophylline-dependent riboswitch ENYC4 (Nakahira et al. 2013), was constructed (Fig. 7a). Cells were first cultivated under the salt stress condition (4% NaCl) for 48 h to accumulate GG. Then theophylline was supplemented to induce *ggp* expression, and the content of GG was monitored (Fig. 7c). Apparent GG degradation induced by theophylline was observed, in particular at high theophylline concentrations. At 750 and 1000 mM of theophylline, the intracellular GG of QL518 was significantly decreased in 48 h, with a reduction of 27.1% and 39.1%, respectively. With regard to the control QL514, it maintained a stable level of GG throughout the testing period in the presence of 1000 mM theophylline. Together, these data demonstrated a clear function of GGP in the degradation of GG in the living cells of cyanobacteria.

## Discussion

*Marinobacter* species are often isolated from marine and other saline environments. They are generally known for the good tolerance to high salinity (up to 20%) (Nie et al. 2021; Yoo et al. 2020; Zhang et al. 2008, 2020a). In agreement with this property, a few pathways for compatible solute syntheses were detected in many isolates of *Marinobacter* (Table S1 and Fig. 2). We found that 53 out of 250 annotated *Marinobacter* genomes contained the putative *ggpPS* gene (Table S1), indicating the potential of GG synthesis under special conditions (e.g., stresses). More interestingly, a *ggp* homolog was always present in associated with *ggpPS* (excluding four cases with partial *ggpPS* at the termini of genomic contigs) and located adjacent to the latter gene. This phenomenon strongly suggested the involvement of *ggp* in GG metabolism. To date, only one pathway, namely GGHA, for microbial GG catabolism was physiologically verified (Kirsch et al. 2017; Savakis et al. 2016). This was done in cyanobacteria. The genetic and biochemical investigations of Syn6803 showed that GG is catabolized by GGHA under low salinity conditions via a hydrolytic process, generating glucose and glycerol. In most of the GG-producing cyanobacteria, the putative *gghA* gene is closely linked with the key GG-synthesizing gene *ggpS*, echoing their metabolic correlation (Fig. 1). However, in the genomes of *Marinobacter*, no *gghA* homologs were detected, indicating the absence of the GGHA pathway. Hence, it becomes logic to suppose a GG-catabolizing role of *ggp* in *Marinobacter*.

Here, detailed investigations on the function of GGP of *M. salinexigens* ZYF650^T^, an isolate from the hadal seawater with a salt tolerance of 0–14% NaCl (Ahmad et al. 2020), were performed. The purified GGP catalyzed apparent decomposition of GG with the products glycerol and glucose, supposing the hydrolysis of GG. However, our quantitative analyses of the products did not support a pure hydrolysis. The amount of glycerol product was significantly higher than that of glucose (Fig. 3d), raising a question that “where is the other part of glucose.” In 2018, Franceus and colleagues provided the first evidence for the function of GGP (Franceus et al. 2018). The *M. adhaerens* HP15 GGP (HP15_2853), which had ever been thought to be a sucrose phosphorylase, was identified as a GG phosphorylase, showing a preferred specificity on GG instead of sucrose. The recombinant GGP catalyzed reversible phosphorolysis of GG with αG1P and glycerol as the products or substrates in vitro. In consideration of the high sequence identity between the *M. salinexigens* ZYF650^T^ and *M. adhaerens* HP15 GGPs, the decomposition of GG in the present study was thought to be a two-step process composed of phosphorolysis and hydrolysis. In line with this hypothesis, an increased generation of αG1P using GG as the substrate under Pi elevation conditions and a production of glucose using αG1P as the substrate under Pi free conditions were detected (Fig. 4c and e).

Although the GG-decomposing activity of *Marinobacter* GGPs has been solidly verified by the previous study as well as the present one (Franceus et al. 2018), the physiological roles of these enzymes are still unclear. Physiological investigations are thereby expected. In the cell, the Pi content is expected to be higher than that of αG1P, because the latter compound can be easily captured by glycolysis. From this perspective, GGP is thought to perform a catabolic function via phosphorolysis in vivo. A phenomenon that may be related with this hypothesis is that, a weak increase of cell density was seen for the wild-type *M. salinexigens* ZYF650^T^ grown in minimal salt medium with GG (0.2 M) as the sole carbon and energy source, while the Δ*ggp* mutant did not show any growth at all (Fig. 6b). To give more information for the function of GGP, physiological analyses were further performed both natively (in *M. salinexigens* ZYF650^T^) and heterologously (in Syn6803) under salinity shift conditions. No matter in the wild-type Syn6803 or the *gghA*-deficient mutant (QL514), the heterologous expression of *ggp* indeed caused a great suppression of the salt-induced GG accumulation (Fig. 7b). A direct depletion of accumulated GG was also observed with the induced expression of GGP (Fig. 7c). Because this analysis was conducted in the *gghA*-deficient background, the influence of the native catabolizing pathway could be eliminated. These results confirmed the GG-catabolizing activity of GGP in the living cells. On the other hand, the analysis in *Marinobacter* was limited because of the failed induction of intracellular GG accumulation. No GG was detected in the wild-type cells of *M. salinexigens* ZYF650^T^ after salt treatment at all tested concentrations (up to 12% NaCl). (i) One explanation can be that GG is not the main compatible solute of this bacterium and stays at a non-detectable level due to the fast turnover. Putative anabolic pathways of ectoine (EctBAC), GB (BetAB), and GGA (GPGS and GPGP), the other three well-identified compatible solutes in many bacteria, were found in 228, 229, and 199 cases of 250 *Marinobacter*S isolates, respectively (Table S1 and Fig. 2). They were more widely distributed among *Marinobacter* than the putative GG metabolic pathways (49/250). However, we also noticed that two results did not support the above conjecture: GG was not accumulated in the wild-type *M. salinexigens* ZYF650^T^ at relatively high NaCl concentrations (such as 9% and 12%, equivalent to 1.5 M and 2.1 M, respectively), at which GGP did not exhibit activity (Fig. 5a); and GG accumulation was also not observed when the *ggp* gene was inactivated (Fig. 6a). (ii) Another possibility is that the tested conditions are not suitable to induce GG synthesis. Many investigations have reported that bacteria produce compatible solutes (e.g., ectoine, 5-hydroxyectoine, trehalose, GGA, and sucrose) under diverse stress conditions such as high salinity, high or low temperature, nitrogen starvation, and desiccation (Alarico et al. 2014; Bursy et al. 2008; Cánovas et al. 2001; de Alvarenga et al. 2020; Kuhlmann et al. 2008). In addition to salt treatment, in the present study, the effect of relatively high temperatures (e.g., 45 °C) was also evaluated (Fig. S4b). Unfortunately, *M. salinexigens* ZYF650^T^ did not produce detectable GG at all these temperatures. Further investigations on more stress conditions need to be performed.

GGP is a GG-specific phosphorylase that belongs to the GH13_18 family in the carbohydrate-active enzyme database (CAZy, http://cazy.org). It is able to catalyze reversible phosphorolysis of GG under proper conditions. In terms of biotechnology, this provides the possibility to synthesize GG by the utilization of the reverse phosphorolytic reaction. Several efforts to produce GG from αG1P and glycerol using *M. adhaerens* HP15 GGP have been conducted (Sun et al. 2023; Zhang et al. 2020b). To reduce production cost, αG1P was provided from low-cost sucrose (or maltodextrin) instead of direct addition by a coupling use of sucrose phosphorylase (or α-glucan phosphorylase). Under the optimized condition, a production of 61 g/l GG was achieved after 24 h using maltodextrin and glycerol as substrates. The results of Sun et al. also demonstrated that the GGPs of different bacterial sources presented distinct activities to catalyze GG synthesis. Hence, the further identification of GGPs will provide more options of enzyme catalysts in GG biosynthesis.

## Supplementary Information

Below is the link to the electronic supplementary material.

Supplementary file1 (PDF 629 KB)

Supplementary file2 (XLSX 36802 KB)

## Data Availability

Data will be made available on request.
