# Supplementary material for: Glucosylglycerol phosphorylase, a potential novel pathway of microbial glucosylglycerol catabolism
Source: Appl Microbiol Biotechnol. 2024 Feb 16;108(1):214. doi: 10.1007/s00253-024-13035-3 (PMC10873239; doi:10.1007/s00253-024-13035-3)
Supplement: Supplementary file 1 — Supplementary file1 (PDF 629 KB) [file 253_2024_13035_MOESM1_ESM.pdf]

(Applied Microbiology and Biotechnology)

Glucosylglycerol phosphorylase, a potential novel pathway of microbial glucosylglycerol catabolism

Lin Cheng[1,2], Zhichao Zhang[2,3,4], Daling Zhu[1], Quan Luo[2,3,4], Xuefeng Lu[2,3,4,5]

[1]College of Chemical Engineering and Materials Sciences, Tianjin University of Science & Technology, Tianjin 300457, China.

[2]Key Laboratory of Biofuels, Qingdao Institute of Bioenergy and Bioprocess Technology, Chinese Academy of Sciences, Songling Rd 189, Qingdao 266101, China.

[3]Shandong Energy Institute, Songling Rd 189, Qingdao 266101, China.

[4]Qingdao New Energy Shandong Laboratory, Songling Rd 189, Qingdao 266101, China.

[5]Marine Biology and Biotechnology Laboratory, Qingdao National Laboratory for Marine Science and Technology, Wenhai Rd 168, Qingdao 266237, China.

Correspondence

Quan Luo; Email: luoquan@qibebt.ac.cn; Tel.: +86-532-80662711

Daling Zhu; Email: zhudaling@tust.edu.cn; Tel.: +86-22-60601305

Table S1 A survey for the metabolic pathways of compatible solutes GG, ectoine, GB, GGA, and sucrose in 250 annotated *Marinobacter* genomes. The query sequences used for the local blastp analyses are given on the top. GGP (HP15_2853) and GGPPS (HP15_2852) are from *M. adhaerens* HP15. GGHA (Slr1670), SPS (Sll0045), SPP (Slr0953), and invertase (Sll0626) are from *Synechocystis* sp. PCC 6803. EctB (HALO_2589), EctA (HALO_2588), EctC (HALO_2590), DoeA (HALO_3665), DoeB (HALO_3664), and DoeD (HALO_3661) are from *H. elongate* DSM 2581^{T}. BetA (CAA37093.1) and BetB (CAA37092.1) are from *E. coli*. GSMT (AAF87202.1) and SDMT (AAF87203.1) are from *Ectothiorhodospira halochloris*. GPGS (SYNPCC7002_A2021), GPGP (SYNPCC7002_A2023), and GGAP (SYNPCC7002_A2022) are from *Synechococcus* sp. PCC 7002. "/" represents a sequence identity of <30%. (The table is given as an electronic Excel file elsewhere.)

Nucleotide sequence The optimized nucleotide sequence of *FWJ25_14990* for heterologous expression in *E. coli* BL21(DE3).

ATGCTGCTGAAAAATGCGGTGCAGCTGATTTGCTATCCGGATCGCATTGGCAATAATCT
GACCGATCTGCATACCGCGGTGGAAAAACATCTGAGCGATGCGATTGGCGGCCTGCAT
ATTCTGCCGTTTTTTCCGAGCAATGCGGATGGCGGCTTTAGCCCGTTAACCCATAAAGA
AGTGGATCCGGCGTTTGGCACCTGGGATGATATTGAAGCGTTTACCGGCAAATATGACC
TGTGCGTGGATCTGACCGTGAATCATATTAGCGATGAAAGCCCGGAATTTCGCGATTTT
ATTGCGAATGGCTTTGACAGCGAATATGCGGATCTGTTTGTGCATGTGGATCGCTTTGG
CGATATTAGCCCGGATGATATGGCGAAAATTCATATTCGCAAAGAGAAGGAGCCGTTCC
GCGAAGTGACCCTGGCGGATGGTACCAAAACCCGCGTTTGGTGTACCTTTACCGAACA
GCAGATTGATCTGAATTATGACGGCGATCTGGCGTATCGCCTGATGGAAAGCTATATTG
GCTTTCTGACCAGCAAAGGCGTGAATCTGCTGCGCCTGGATGCGTTTGGCTATACCACC
AAACGCATTGGCACCAGCTGCTTTCTGGTGGAACCGGAAGTGTATCGCATTCTGGATT
GGATTAATGAGGTGGCGTTTAAACACGGCGCGGAATGCCTGCCGGAAGTGCATGATCA
TACCAGCTATCAGTATGCGATTAGCCGCCGCAATATGCATCCGTATGGCTTTGCGCTGCC
GCCGTTATTACTGTATAGCCTGCTGGATGCGAATAGCGTGTATCTGAAAAATTGGCTGC
GCATGTGCCCGCGCAATATGGTGACCGTTTTGGATACCCATGATGGCATTTGCATTCCG
GATGTGGAAGGCGTGCTGCCGGATGATAAAATTAAAGTGCTGATTGACAACATCGACG
CGCGCAGCGCGGATCCGATTATGAGAAGAAGCGCGGCGAATATTCATAGCGTGGGCGC
GATTTATCAGCTGACCTGCACCTTTTATGATGCGCTGATGCAGAATGATGATGCGTATAT
TGCGGCGCGCGCGATTCAGTTTTTTACCCCGGGTATTCCGCAGGTGTATTATGTGGGCC
TGCTGGCGGGTTGCAATGATCAGGAACTGATGGAAAAAACCGGCGAACTGCGCGATAT
TAATCGCAATTATTATACCCTGAACGAGATGGACGAAGCGATGGAAAAACCGGTGGTG
CAGCGCCTGTTAACCTTGATGAAATTTCGCACCAATTATCCGGCGTTTGATGGCCATTTT
GAACTGAATTATAGCAACGACAGCAGCGTGGCGATGGCGTGGCGTCATGGTGAACATT
ATTGCCATCTGTTTGTGGATCTGAATTTCAACACCAGCAAAATCCAGTACGTGGACGTG
AAAAGCGGCGAAACCCGCGATCTGGAATTTTAA

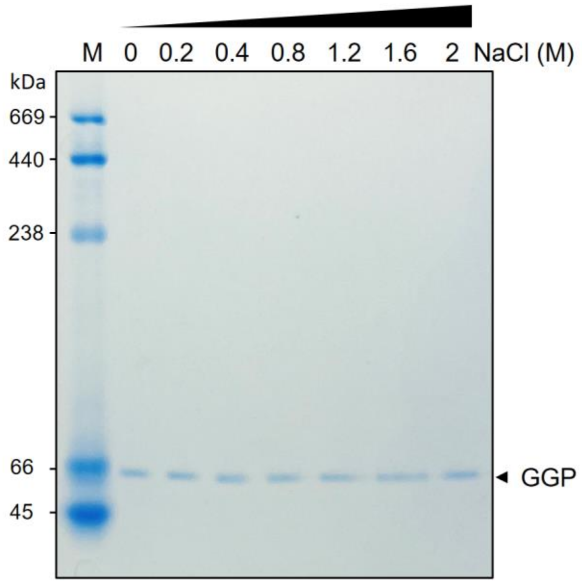

Fig. S1 Analysis of the impact of salt treatment on purified GGP by native PAGE. The purified GGP protein (0.05 mg/ml) was incubated in 20 mM PB buffer (pH6.6) supplemented with different NaCl concentrations (0-2 M as indicated) at 45°C for 0.5h. A proper amount of mixture was analyzed by native PAGE. The solid triangle indicates the protein band of GGP. M, protein marker.

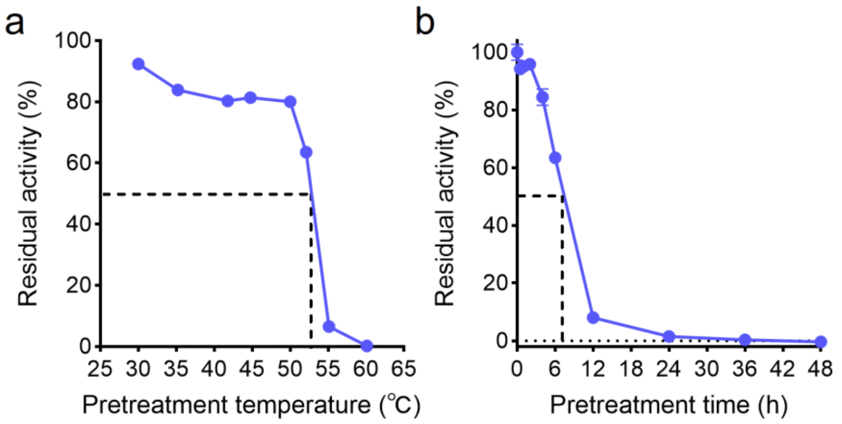

Fig. S2 Analysis of the thermostability of GGP. In (a), the purified GGP was incubated at 30-60℃ for 10 min and then used for enzyme assays. In (b), the purified GGP was incubated at 45℃ for 0-48 h and then used for enzyme assays. All values were normalized by comparing them with the control values (no heat treatment). The dashed lines were used to assist in determining the semi-inactivation temperature and half-life time of GGP.

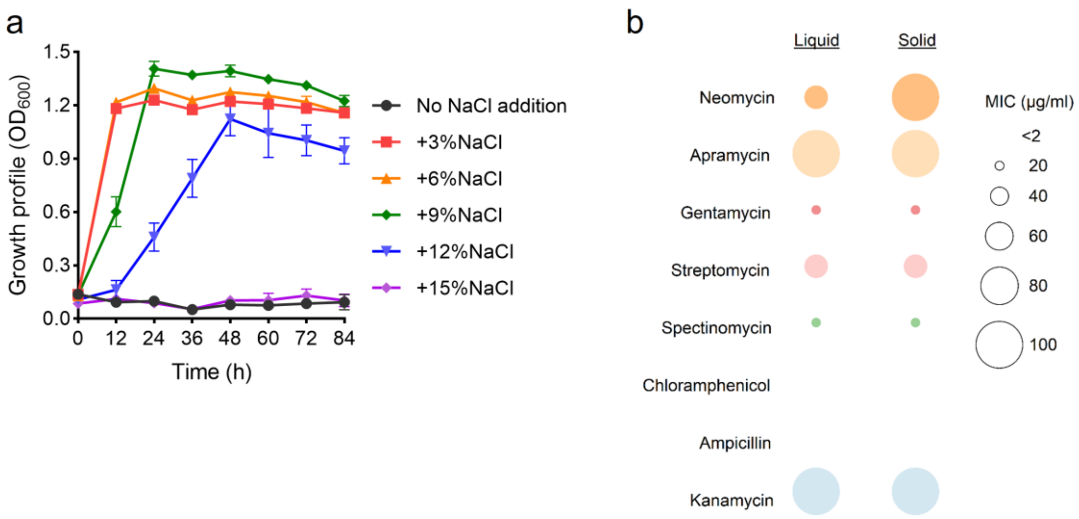

Fig. S3 Analyses of the salt tolerance (a) and antibiotic sensitivity (b) of the wild-type *M. salinexigens* ZYF650^T. In (a), cells were grown in liquid MB medium containing 0-12% NaCl. In (b), cells were cultivated in liquid or on solid MB medium (containing 6% NaCl) with different concentrations of kanamycin, ampicillin, chloramphenicol, streptomycin, spectinomycin, gentamycin, apramycin, and neomycin, respectively.

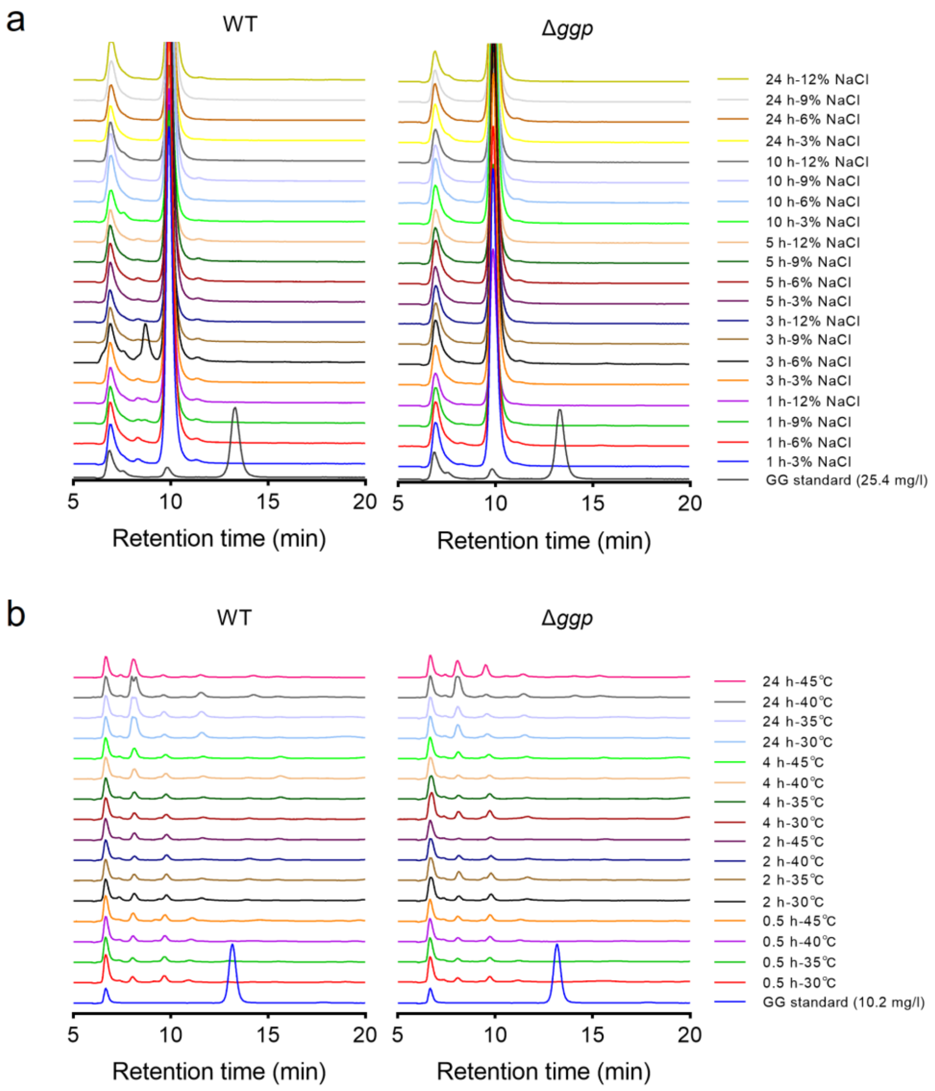

Fig. S4 Analysis of the possible GG accumulation of the *M. salinexigens* ZYF650^T wild-type (WT) and Δ*ggp* strains under different NaCl and temperature conditions. In (a), *Marinobacte*r cells were grown in minimal salt medium (5.3 g/l $MgCl_2$-$6H_2O$, 0.75 g/l KCl, 0.1 g/l $MgSO_4$-$7H_2O$, 50 mg/l K_2HPO_4, 1 g/l NH_4Cl, 0.74 g/l $CaCl_2$-$2H_2O$, 0.42 g/l $NaHCO_3$, and 20 g/l NaCl) containing 0.5% (w/v) glycerol for 24 h. NaCl was supplemented to the final concentrations as indicated. The residual glycerol in the samples showed a retention time of ~10 min. In (b), cells were grown in MB medium containing 6% NaCl at 30-45°C.
